# Supplementary material for: Antimicrobial Susceptibility Profiles of Bacteria Commonly Isolated from Farmed Salmonids in Atlantic Canada (2000–2021)
Source: Vet Sci. 2022 Mar 25;9(4):159. doi: 10.3390/vetsci9040159 (PMC9031543; doi:10.3390/vetsci9040159)
Supplement: Supplementary file 1 [file vetsci-09-00159-s001.zip › vetsci-1641290-supplementary.pdf]

Antimicrobial Susceptibility Profiles of Bacteria Commonly Isolated from Farmed Salmonids in Atlantic Canada (2000–2021)

Rasaq Abiola Ojasanya [1,*], Ian A.Gardner [1], David B. Groman [2], Sonja Saksida [1], Matthew E. Saab [2] and Krishna Kumar Thakur [1]

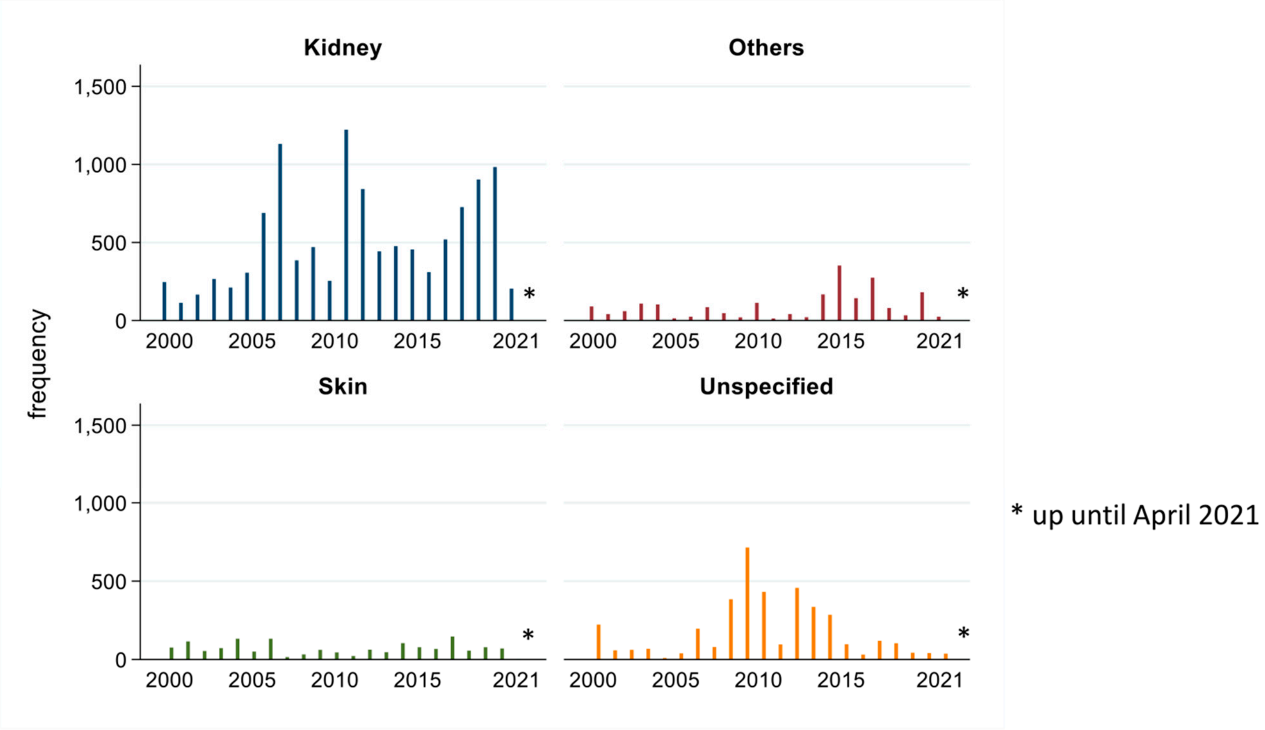

Figure S1. Annual frequency of salmonid samples submitted for bacteriology to AVC ADSBL from 2000 to 2021 by anatomic site of sample collection.

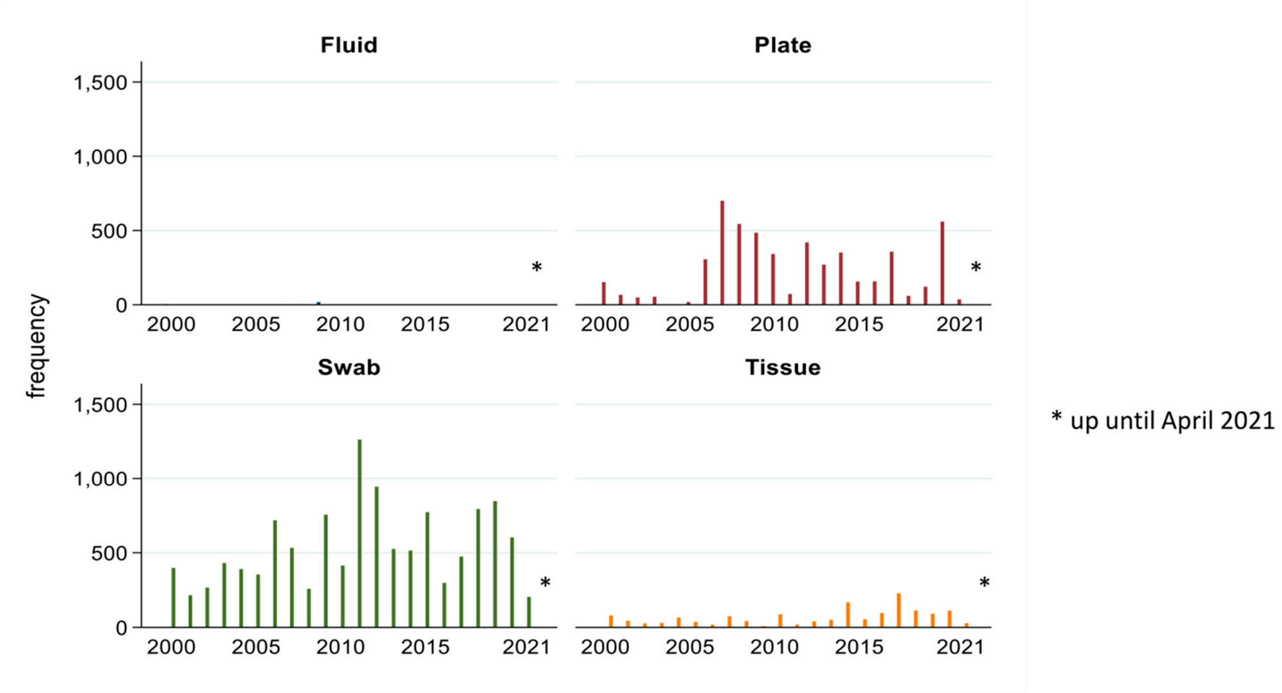

Figure S2. Annual frequency of salmonid specimens submitted for bacteriology to AVC ADSBL from 2000 to 2021.

Table S1. Frequency of samples originating from different anatomic sites submitted to the AVC ADSBL (2000-2021).

Species	Kidney (n = 11,320)	Skin (n = 1517)	Unspecified (n = 3905)	Others (n = 2034)
n (%)				
Atlantic salmon	9994 (88.29)	1392 (91.76)	3700 (94.75)	1782 (87.61)
Rainbow trout	1326 (11.71)	125 (8.24)	205 (5.25)	252 (12.39)

Table S2. Bacterial isolates detected from different anatomic sites of salmonids samples submitted to the AVC ADSBL (2000-2021).

Bacterial Isolates	Kidney (n = 11,320)	Skin (n = 1517)	Unspecified (n = 3905)	Others (n = 2034)
	%			
Mixed microbiota	9.5	23.7	17.4	17.4
Gram-negative bacilli	5.4	7.0	16.8	16.8
Vibrio spp.	5.6	15.2	8.9	7.0
Pseudomonas spp.	2.4	2.8	3.2	4.9
Aeromonas spp.	2.2	2.9	2.4	3.2
Flavobacterium spp.	0.3	1.9	0.6	4.7
Gram-positive bacilli	1.9	0.7	2.4	2.4
Yersinia spp.	1.4	0	1.1	1.1
Vibrio anguillarum	0.1	0	1.2	1.2
Mycobacterium spp.	0.3	0.1	1.2	0.1
Aeromonas sobria	0.3	0.1	0.6	0.6
Edwardsiella piscicida	1.4	0	0	0
Gram-positive bacteria	0.1	0	0.5	0.5
Arthrobacter spp.	0.4	0	0.3	0.3
Enterobacter spp.	0	0.5	0.2	0.2
Serratia spp.	0.1	0	0.4	0.4
Aliivibrio salmonicida	0.5	0	0.2	0.2
Micrococcus spp.	0	0	0.3	0.3
Shewanella putrafaciens	0	0.5	0	0
Moraxella spp.	0	0.1	0.2	0.2
Vibrio alginolyticus	0	0	0.2	0.2
Bacillus spp.	0	0	0.3	0.3
Staphylococcus spp.	0.1	0	0.1	0.1
Rhodococcus spp.	0.1	0	0.1	0.1
Lactobacillus spp.	0.1	0	0.1	0.1
Hafnia alvei	0.2	0	0	0
Plesiomonas spp.	0.2	0	0	0

Staphylococcus epidermis	0	0	0.1	0.1
Streptococcus spp.	0	0	0.1	0.1
Yersinia ruckeri type 2	0.2	0	0	0
Aliivibrio fisherii	0	0.1	0	0
Carnobacterium maltaromaticum	0.1	0	0	0
Enterococcus spp.	0.1	0	0	0
Photobacterium spp.	0.1	0	0	0
Proteus mirabilis	0.1	0	0	0
Proteus spp.	0.1	0	0	0
Pseudomonas putida	0.1	0	0	0
Yersinia ruckeri	0.1	0	0	0
Corynebacterium spp.	0	0.1	0	0

n = number of isolates, Unspecified- sample sites without clear identification. Others-include sample sites from the abdominal cavity, blood, bladder, brain, ova, eye, fin, gill, head, heart, intestine, jaw, liver, mouth, muscle, ovarian fluid, peritoneal cavity, spleen, tail, vent, wound, and yolk sac.

Table S3. Frequency of bacterial isolates detection in submitted cases to the AVC ADSBL by species of salmonid samples (2000-2021).

**Bacterial Isolates**	**Frequency of Cases (n)**	**Atlantic Salmon n (%)**	**Rainbow Trout n (%)**
Mixed microbiota	300	281 (93.7)	19 (6.3)
Gram-negative bacilli	284	269 (94.7)	15 (5.3)
Vibrio spp	150	145 (96.7)	5 (3.3)
Gram-positive bacilli	54	47 (87.0)	7 (13.0)
Aeromonas spp.	35	34 (97.1)	1 (2.86)
Mycobacterium spp.	19	19 (100.0)	0
Flavobacterium spp.	14	11 (78.57)	3 (21.4)
Yersinia spp.	12	12 (100.0)	0
Nocardia spp.	13	13 (100.0)	0
Gram-positive cocci	9	6 (66.7)	3 (33.3)
Gram-negative bacteria	9	5 (55.6)	4 (44.4)
Bacillus spp.	5	5 (100.0)	0
Gram-positive bacteria	5	5 (100.0)	0
Lactobacillus spp.	4	4 (100.0)	0
Rhodococcus spp.	4	4 (100.0)	0

Serratia liquefaciens	4	4 (100.0)	0
Staphylococcus spp.	4	4 (100.0)	0
Vibrio anguillarum type 2	4	4 (100.0)	0
Yersinia ruckeri type 2	3	3 (100.0)	0
Enterobacter spp.	3	3 (100.0)	0
Plesiomonas spp.	3	3 (100.0)	0
Carnobacterium maltaromaticum	2	1 (50.0)	1 (50.0)
Aliivibrio fischeri	2	2 (100.0)	0
Gram-positive coccobacillus	2	2 (100.0)	0
Micrococcus spp.	2	2 (100.0)	0
Acinetobacter spp.	1	1 (100.0)	0
Chryseobacterium spp.	1	1 (100.0)	0
Lactococcus lactis	1	0	1 (100.0)
Photobacterium damsel	1	0	1 (100.0)
Photobacterium spp.	1	0	1 (100.0)
Proteus vulgaris	1	1 (100.0)	0
Pseudomonas chlororaphis	1	1 (100.0)	0
Pseudomonas putida	1	1 (100.0)	0
Serratia spp.	1	1 (100.0)	0
Staphylococcus epidermidis	1	1 (100.0)	0
Streptococcus spp.	1	1 (100.0)	0
Corynebacterium spp.	1	0	1 (100.0)
Vibrio anguillarum	1	1 (100.0)	0
Vibrio fischeri	1	1 (100.0)	0

Table S4. Antimicrobial susceptibility patterns (% S, I, and R) for tested antimicrobials in bacterial isolates from samples of salmonid. (2000-2021)

		**Florfenicol**				**Oxytetracycline**				**Ormetoprim-sulfadimethoxine**				**Trimethoprim-sulfamethoxazole**		
**Bacterial Isolates**	**N**	**n**	**S**	**I**	**R**	**n**	**S**	**I**	**R**	**n**	**S**	**I**	**R**	**n**	**S**	**I**	**R**
Vibrio spp.	141	140	100.0	0	0	141	91.0	2.1	7.1	63	45.0	0	0	138	97.2	0	0.7
Pseudomonas spp.	53	53	7.6	9.4	83.0	53	70.0	15.1	15.1	7	0	0	13.2	53	22.6	11.3	66.0
Aeromonas spp.	45	44	91.1	2.2	4.4	44	36.0	2.2	60.0	6	13.3	0	0	44	91.1	0	6.7
Yersinia spp.	28	27	100.0	0	0	28	96.0	0	3.6	25	3.9	0	0	28	100.0	0	0

Flavobacterium spp.	21	21	85.7	0	14.3	21	67.0	0	33.3	3	9.5	0	4.8	18	57.1	0	28.6
Nocardia spp.	12	12	8.3	42.0	50.0	12	8.3	25.0	66.7	5	41.7	0	0	12	100.0	0	0

		**Enrofloxacin**				**Ampicillin**			
**Bacterial Isolates**	**N**	**n**	**S**	**I**	**R**	**n**	**S**	**I**	**R**
Vibrio spp.	141	141	100.0	0	0	141	48.9	4.3	46.8
Pseudomonas spp.	53	50	86.8	7.6	0	53	1.9	0	98.1
Aeromonas spp.	45	41	91.1	0	0	44	6.7	0	91.1
Yersinia spp.	28	28	100.0	0	0	28	64.3	3.6	32.1
Flavobacterium spp.	21	21	100.0	0	0	21	23.8	4.8	71.4
Nocardia spp.	12	12	100.0	0	0	12	0	0	100.0

N= Total number of isolates, n=number of isolates that were tested for antimicrobial susceptibility, S- Susceptible, I- Intermediate, R- Resistant cumulative percentages may not add up to 100 as not all isolates had susceptibility test.
